# The impact of COVID-19 self-isolation on healthcare workers’ psychological wellbeing: a systematic review

**DOI:** 10.1093/occmed/kqaf124

**Published:** 2025-12-31

**Authors:** M V Stein, S K Brooks, L E Smith, G J Rubin, R Amlôt, N Greeberg, A F Martin

**Affiliations:** Department of Psychology, Institute of Psychiatry, Psychology & Neuroscience, King’s College London, 16 De Crespigny Park, London, SE5 8AB, UK; Department of Psychological Medicine, Institute of Psychiatry, Psychology & Neuroscience, King’s College London, Weston Education Centre, Cutcombe Road, London, SE5 9RJ, UK; UK Health Security Agency, 10 South Colonnade, Canary Wharf, London, E14 4PU, UK; Department of Psychological Medicine, Institute of Psychiatry, Psychology & Neuroscience, King’s College London, Weston Education Centre, Cutcombe Road, London, SE5 9RJ, UK; UK Health Security Agency, 10 South Colonnade, Canary Wharf, London, E14 4PU, UK; Department of Psychological Medicine, Institute of Psychiatry, Psychology & Neuroscience, King’s College London, Weston Education Centre, Cutcombe Road, London, SE5 9RJ, UK; Department of Psychological Medicine, Institute of Psychiatry, Psychology & Neuroscience, King’s College London, Weston Education Centre, Cutcombe Road, London, SE5 9RJ, UK

## Abstract

**Background:**

Self-isolation is a key public health strategy for infectious disease control. Globally implemented during the COVID-19 pandemic, self-isolation remains an essential strategy in ongoing mitigation efforts. Healthcare workers (HCWs) often face isolation due to occupational exposure to infectious diseases and may face unique psychological challenges.

**Aims:**

This systematic review synthesized evidence on (i) the impact of self-isolation on HCWs’ psychological wellbeing, (ii) factors associated with wellbeing, and (iii) the effectiveness of interventions to improve wellbeing during or after isolation for COVID-19.

**Methods:**

A pre-registered systematic review (PROSPERO: CRD42024559971) was conducted in accordance with PRISMA (Preferred Reporting Items for Systematic Reviews and Meta-Analyses) and Cochrane guidelines. Searches in PsycInfo, Embase, MEDLINE, PubMed and grey literature included studies on HCWs’ psychological wellbeing during or after self-isolation. Risk of bias was assessed using ROBINS-E (Risk of Bias in Non-randomized Studies for Exposure) or CASP (Critical Appraisal Skills Programme) tools.

**Results:**

From 20,798 records screened, 19 studies (10 quantitative, 7 qualitative, 2 mixed methods) were included. Quantitative findings on anxiety, depressive and stress symptoms were inconsistent. Qualitative studies consistently reported distress, loneliness and stigma. Factors associated with wellbeing included socio-cultural influences and protective factors. No studies assessed interventions targeting wellbeing during self-isolation.

**Conclusions:**

Self-isolation appears to have variable effects on HCWs’ wellbeing, including significant challenges and opportunities for resilience. Public health strategies should prioritize timely, clear communication, accessible evidence-based psychological support and practical resources. Future research should prioritize evaluation of interventions to mitigate psychological harm and support HCWs during infectious disease outbreaks.

Key learning points

**What is already known about this subject:**

- Self-isolation is a widely implemented public health strategy for infectious disease control, yet its psychological impact on healthcare workers (HCWs), who face unique occupational risks, remains poorly understood.
- Prior evidence on self-isolation’s psychological effects has largely focused on the general population, with limited synthesis of its specific impact on HCWs’ wellbeing.
- The absence of systematic evaluation of wellbeing outcomes or supportive interventions for HCWs during isolation highlights a knowledge gap relevant to ongoing and future public health emergencies
- **What this study adds:**
- This review identifies that self-isolation has variable psychological impacts on HCWs, with qualitative studies consistently reporting distress, loneliness and stigma, while quantitative findings were inconsistent and limited by methodological concerns.
- It highlights key factors influencing wellbeing, including cultural norms, workplace dynamics and social support, as well as protective features such as resilience and post-traumatic growth.
- Importantly, the study reveals a complete absence of evaluated interventions to support HCWs’ mental health during self-isolation, highlighting the need for targeted, evidence-based approaches.
- **What impact this may have on practice or policy:**
- Occupational health policy should address psychological risks associated with mandated self-isolation, incorporating timely communication, mental health resources and stigma reduction strategies.
- Institutional preparedness plans should integrate proactive psychological support for HCWs during infectious disease outbreaks, recognizing self-isolation as a period of elevated vulnerability.
- This review informs the development of evidence-based interventions and highlights the urgency of embedding mental health support within infection control protocols.

## INTRODUCTION

During the COVID-19 pandemic, isolation and quarantine were globally implemented public health strategies to control the spread of the virus. Isolation is the separation of those who are sick from those who are well; while quarantine is the separation of those who have been exposed to an illness but are not yet symptomatic. Both measures are critical strategies to curb the transmission of infectious diseases [1]. For the purpose of this review the term ‘self-isolation’ encompasses both isolation and quarantine, distinct from broader population-wide ‘lockdowns’. A recent review by Martin *et al.* [2] showed that self-isolation was associated with generally negative mental health and wellbeing outcomes for individuals in the general population, particularly post-traumatic stress disorder (PTSD).

Healthcare workers (HCWs), in the present review defined as pre-licensed or licensed physical and mental health professionals, hospital staff and administrators, were particularly vulnerable to infection during the COVID-19 pandemic due to their occupational exposure [3]. The nature of their roles often placed them at significantly higher risk of contracting the virus [4]. As such, many healthcare settings implemented self-isolation protocols, especially for HCWs directly exposed to infected individuals, in order to mitigate the spread of infection [5]. This included post-shift self-isolation requirements either at home or in a work-sanctioned isolation facility (e.g. hospital ‘dorms’ or contracted hotels), regardless of whether the individual had been in direct contact with an infected person.

The combination of occupational exposure and self-isolation protocols introduced challenges for HCWs, compounding the psychological burden they faced during the pandemic [2,6,7] arising from exposure to occupational trauma, increased risk of infection, repeated periods of self-isolation and a heightened fear of transmitting the virus to family members [8–10]. Consequently, HCWs may have experienced distinct psychological outcomes compared to the general population, including moral injury, trauma and burnout [11,12]. Understanding these unique stressors and outcomes is essential for shaping future public health strategies, particularly as self-isolation remains a key measure in managing infectious diseases (e.g. 2022 mpox outbreak) [8] and for the treatment of diseases that present a significant burden globally, such as tuberculosis [13].

To address these gaps in the literature, we conducted a pre-registered systematic review with three primary aims: (i) to synthesize and evaluate the impact of self-isolation on psychological and emotional wellbeing (hereafter wellbeing) of HCWs during the COVID-19 pandemic; (ii) to identify factors associated with wellbeing outcomes in HCWs during or after self-isolation; and (iii) to assess the effectiveness of interventions designed to improve the wellbeing of HCWs during or following self-isolation. To achieve these aims, we systematically identified and appraised original studies, narratively synthesizing their findings to provide a comprehensive overview of the current evidence.

## METHODS

We completed this prospectively registered review (PROSPERO registration number: CRD42024559971) in accordance with Cochrane Collaboration guidelines [14] and the Preferred Reporting Items for Systematic Reviews and Meta-Analyses (PRISMA) [15]. We deviated from our pre-registration and did not extract specific effect sizes or measures of precision (e.g. standard errors or confidence intervals) from included studies.

The papers subjected to synthesis must have been published on or after 1 January 2020 and contain original data relating to the psychological wellbeing of HCWs during or after COVID-19-­related self-isolation. HCWs may be identified as the main population of interest, or within a broader study where data from HCWs are reported separately from that of the general population. We excluded studies where HCWs were placed into isolation in a hospital or healthcare setting for treatment of COVID-19, rather than as a precaution to prevent the spread of COVID-19. For studies that assessed the impact of self-isolation (Aim 1), they must have employed a pre-exposure comparison (baseline, within-subjects) or control group comparison (non-isolated, between-groups). In studies that assessed factors associated with wellbeing outcomes (Aim 2), we included studies that looked at factors occurring during (e.g. communication quality, individual difference factors, etc.) or related directly (e.g. duration of isolation) to the self-isolation period. In studies that assessed the efficacy of an intervention to improve wellbeing (Aim 3), the study design must have reported pre- and post-intervention scores in the intervention group and/or a control group comparison.

Medline, PsycInfo, Web of Science, Embase, PsyArXiv and medRxiv were systematically searched. An initial search was performed in December 2022 by A.F.M., the search was repeated in August 2023 and May 2024 by M.V.S., resulting in two new studies for inclusion. The search string (see https://tinyurl.com/ymy363k2) was augmented with manual searches of all included articles’ reference lists and relevant reviews captured in the initial search. We previously searched five grey literature databases for intervention studies only [2] but did not update this search for the present review, as no studies were identified through this method from previous searches.

The initial screening was conducted as part of a complementary review looking at wellbeing in the adult population [2]. In the previous review, the search was piloted, and the screening team (A.F.M., L.E.S., S.K.B., M.V.S., and G.J.R.) reviewed a training set of 300 studies. Discrepancies were discussed until agreement on included studies was attained. During the formal study selection process, one team member (A.F.M.) set aside articles that met the current review’s inclusion criteria and confirmed study selection with the screening team. The authors of three potentially eligible papers were contacted for methodological clarification; these papers were excluded because they did not meet the inclusion criteria.

Data extraction was performed by one reviewer (M.V.S. for quantitative and S.K.B. for qualitative), all extracted data are available open access (see https://osf.io/jngme/). During data extraction, any uncertainties were discussed with the screening team. The data subjected to extraction were defined *a priori* and included: (i) study characteristics: (a) country, (b) dates of data collection, (c) study design and data collection method, (d) sampling details, (e) inclusion criteria and final sample details (age, sex, etc.); (ii) isolation characteristics: (a) reason and location of self-isolation, (b) assessment points, (c) temporal proximity of data collection to self-isolation period; (iii) quantitative outcomes by research aim: (a) outcome measure, (b) analysis type, (c) significant associations and no evidence for significant associations; and (iv) qualitative outcomes by research aim: (a) measure, (b) analysis method, (c) impact of isolation, (d) factors perceived to be associated; v, other potentially relevant information.

Study quality was assessed by the same two reviewers for each study type. Quantitative study quality was assessed using either the Risk of Bias in Non-randomized Studies for Exposure (ROBINS-E) [16] or Interventions (ROBINS-I) [17]. Papers reporting data addressing more than one of our review’s aims received a ROBINS score for each aim. Studies were rated as low risk, some concerns, high risk or very high risk based on the tool’s algorithm. Qualitative studies were assessed using a modified version of the Critical Appraisal Skills Programme (CASP) checklist, a 10-item measure of study quality [18]. The item ‘how valuable is the research?’ was reworded to ‘do the authors discuss the value of the research in terms of implications and contribution to literature?’. This allowed for binary responses (Yes = 1, No = 0) in line with the other items. An overall quality percent score was computed for each study, with higher scores indicating better quality.

Given the research aim of appraising the significance and direction of the relationship between self-isolation and wellbeing, effect sizes were not reported. To capture effects across diverse methodologies, we prospectively planned to narratively synthesize the results following guidance from Chapter 12 of the Cochrane Handbook [19]. Quantitative studies were narratively synthesized in accordance with synthesis without meta-analysis (SWiM) guidelines [20]. Qualitative studies were synthesized using meta-ethnography following eMERGe guidelines [21]. Syntheses were partitioned *a priori* by review aim and grouped by psychological outcome, further grouping categories were defined *post hoc* as part of the process of synthesis.

## RESULTS

**Figure 1.**
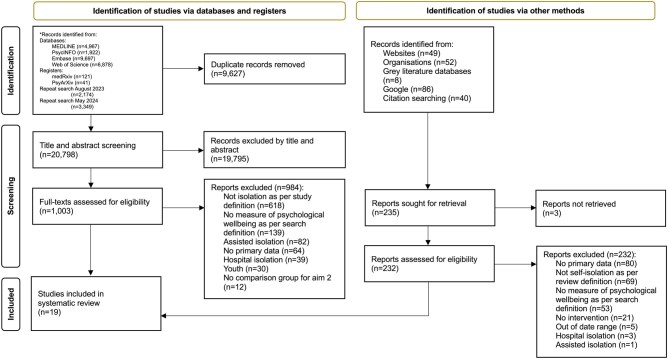
PRISMA flowchart of the study selection process.

A PRISMA diagram presenting study selection can be found in Figure 1. The final sample included 19 papers, the details of which are summarized in Table 1. A full list of included papers is available in the open access data file ( https://osf.io/jngme/). A total of 18 studies reported findings related to the impact of self-isolation on the wellbeing of HCWs (Aim 1). These included nine quantitative studies, seven qualitative studies, and the qualitative components of two mixed-methods studies. Fourteen studies reported factors associated with wellbeing during or after self-isolation (Aim 2). These included three quantitative studies, one of which addressed this aim exclusively, as well as the quantitative components of two mixed-methods studies. Additionally, five qualitative studies and the qualitative components of two mixed-methods studies addressed Aim 2. No studies investigated the effectiveness of interventions aimed at improving the wellbeing of HCWs during or after self-isolation (Aim 3).

**Table 1.** Principal features of included quantitative and qualitative papers

**Source**	Country	Dates of data collection	Healthcare discipline	Quarantine location	*n* (% female)	Age (as reported)	Wellbeing measure	Synthesis aim addressed by study
Alfaifi 2022	Saudi Arabia	June-September 2020	HCW, medical staff working	5-Star hotel	301 (37)	– (–)	DASS-21	1, 2
Alshehri 2021	Saudi Arabia	–	–	Home, hospital, hotel	404 (54)	36.9 (8.7)	PCL-5, Study specific PTSD measure	1
Chan 2022^a^	Singapore	Mid-July–mid-August 2020	Doctors, nurses, allied staff, administrative staff	Home, staff accommodation	663 (76)	21–30 = 30.9%, 31–50 = 56.4%, >51 = 12.7%	Qualitative wellbeing	1,2
Chatterjee 2021	India	April 20–May 20	Doctors, nurses, non-clinical staff members	–	65 (56)	37.67 (9.84)	PSS-10, ISI-7	1
Chew 2022^a^	Malaysia	June 2020	–	–	11 (–)	–	Qualitative wellbeing	1, 2
Demerdash 2021	–	April–November 2020	–	–	65 (43)	Range 26–38 (median: 31)	PSS	1
Dharra 2021	–	–	Nurses	–	368 (58)	28.91 (3.68)	GAD-7, GSE	1
Duong-Quy 2022	Vietnam	–	–	–	100 (86)	18–25 = 88%, 26–24 = 11%, >46 = 1%	4-item sleep scale	1, 2
Fawaz 2020^a^	Lebanon	–	Doctors, nurses, technicians, social worker	Rented apartments near hospital, hospital dormitory	13 (81)	–	Qualitative wellbeing	1
Garcia-Sierra 2021^a^	Spain	September 2020	–	Home	109 (–)	–	Open-ended survey question	1
HaGani 2022	Israel	October–November 2020	Doctors, nurses, paramedical and administrative staff	–	148 (70)	–	GHQ-12, five-item Likert of needs, personal resources and workplace satisfaction	2
Han 2023^a^	Canada	–	Student physicians	–	28 (48)	<26 y = 52.2%, aged 26 or over = 34.8%, not reported = 13%	Survey, interviews	1,2
Holmes 2023^b^	Australia	April–March 2021	–	Home, hotel	106 (75)	18–30 = 42%, 31–40 = 27%, 41–50 = 13%, >50 = 17%	CD-RISC-2, GAD-7, PHQ-9, IES-6, FACIT, CAS, CRS	1,2
Kim 2021	USA	20 April–10 May 2020	Registered nurses	–	320 (83)	Range: 21–67	GAD-7, PHQ-9	1
Moghimian 2022	Iran	June 2020–November 2020	Doctors, nurses	–	19 (63)	42.4 (–)	Qualitative wellbeing	1, 2
Rachubinska 2022	Poland	1 January–1 April 2021	Doctors, nurses, other-HCW	–	207 (83)	37.87 (–)	AIS, IES-R, GAD-7, PSS-10	1
Sagaltici 2022	Turkey	1 April–1 May 2020	–	–	253 (60)	33.57 (8.39)	MBI	1
Venkatesh 2021^a^	India	Mid-July–Mid-September 2020	Doctors, nurses, technicians, social workers, administrators	–	13 (46)	30.1 (–)	Qualitative wellbeing	1, 2
Yin 2024^b^	China	20 October–3 November 2022	Hospital pharmacists	Home	210 (75)	18–30 = 42%, 31–40 = 27%, 41–50 = 13%, >50 = 17%	CD-RISC-2, GAD-7	1, 2

– = Not reported (all instances reflect missing data; NA was never applicable); DASS-21, Depression, Anxiety, and Stress Scale-21; PSS-10, The Perceived Stress Scale; ISI-7, Insomnia Severity Index; PSS, Perceived Stress Scale; GAD-7, The Generalized Anxiety Disorder Scale-7; GSE, The General Self-Efficacy Scale; GHQ-12, General Health Questionnaire-12; PHQ-9, The Patient Health Questionnare-9; AIS, Athens Insomnia Scale; IES-R, Impact of Event Scale-Revised; MBI, Maslach Burnout Inventory; CD-RISC-2, Connor–Davidson Resilience Scale; IES-6, Impact of Life Events Scale; CAS, Coronavirus Anxiety Scale; CRS, Coronavirus Reassurance Seeking Scale.

a Qualitative study.

b Mixed methods.

**Figure 2.**
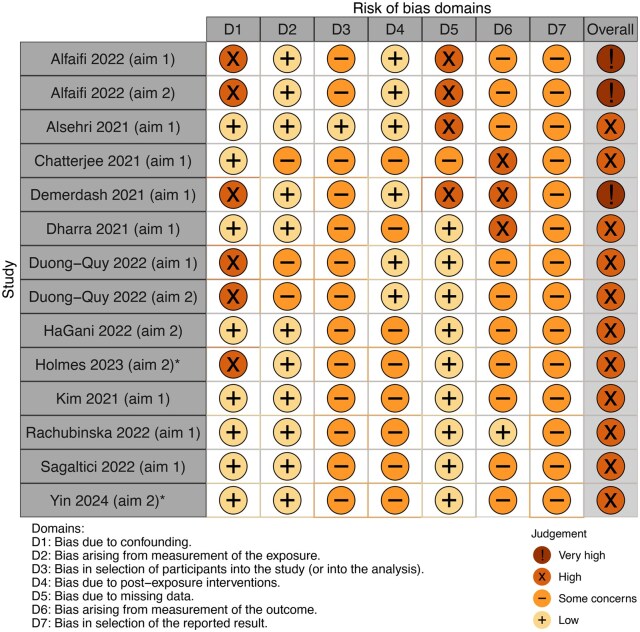
Summary of risk of bias in quantitative studies assessed with the ROBINS-E. *Mixed-methods quantitative result*;* Traffic-light plot was created using robivs web application using colour-blind accessible formatting.

The quantitative impact of self-isolation on wellbeing was reported in nine studies. Two studies were ‘very high risk of bias’ and the rest were ‘high risk’ (Figure 2). Evidence for any impact of self-isolation on wellbeing was variable. Three studies reported a significant association between anxiety symptoms and self-isolation [22–24], while two studies found no significant association [25,26]. Similarly, evidence regarding depression was inconsistent: two studies identified a significant association with self-isolation [22,26], whereas one study did not [24]. Stress was assessed in three studies: one study found that stress levels were higher during self-isolation [27] while two studies found no association [24,25]. Quarantine was found to improve sleep quality in one study [28] and decrease it in another [25]. Other psychological outcomes, such as burnout [29], hopelessness [25], and PTSD symptoms [30] were assessed in a single study. From the studies that were not synthesized, burnout was significantly associated with self-isolation, whereas hopelessness was not; the association with PTSD symptoms was not reported. These single-study findings should be interpreted with caution given the ‘high risk’ of bias across studies.

Nine qualitative studies investigated the impact of self-isolation on wellbeing. Overall, the methodological quality of qualitative studies was satisfactory, on average all studies met over half of the assessed quality criteria (mean ± SD: 73% ± 10%; range: 60–90%). The qualitative findings revealed a wide range of negative psychological and emotional effects experienced by participants during self-isolation. Emotional distress was often reported, with individuals describing feelings of loneliness, sadness, anxiety, tension and general distress [31–36]. Participants expressed fears related to the pandemic, including fears of contracting COVID-19 or potentially spreading it to others, fears of death and fears of being judged or stigmatized [32,33,37–39]. Worries about the health of family and friends, along with financial concerns, were also significant sources of stress [32,34,37].

The qualitative findings also identified positive effects of self-isolation. Some participants described having time for rest and relaxation [34,37], a renewed sense of gratitude, and an appreciation for small joys in life, with some feeling they had been given a second chance [33,37]. Others highlighted quick adaptation to the situation [35] and newfound perspectives on their professional roles, including increased empathy for patients and a stronger sense of professional commitment [32,37].

Factors associated with wellbeing were reported in four quantitative and seven qualitative studies. There was substantial heterogeneity in reported outcomes across methodologies assessing factors associated with wellbeing. Factors associated with wellbeing spanned a broad range of socio-cultural and psychological domains, including risk factors for adverse wellbeing outcomes and factors that protect wellbeing. One quantitative finding was ‘very high risk of bias’ and the rest were ‘high risk’ (Figure 2). As with Aim 1, qualitative findings were of satisfactory quality (mean ± SD: 75% ± 10%; range: 60–90%).

In the quantitative studies, greater COVID-19 stigma was associated with worse wellbeing outcomes [22,40]. Similarly, longer quarantine duration was associated with worse wellbeing outcomes in two studies [38,40], while one study did not find a significant relationship [22]. Finally, bed quality was associated with sleep improvement in one study [28].

In the qualitative studies, participants discussed specific situations and characteristics which they believed made self-isolation more difficult. Having recently moved and still adapting to new circumstances was reported to make self-isolation harder [31], while experiencing other emergencies during the self-isolation period (e.g. being unable to take an unwell child to the doctor) negatively affected mental state [35]. Receiving conflicting information contributed to uncertainty [32]. The perception of being stigmatized by others made the situation more distressing [31]. Perceiving the mitigation measures to be too strict also appeared to negatively affect wellbeing [33]. Inadequate basic supplies in quarantine and/or unmet medical needs also made the situation more difficult to cope with [35,38]. Other challenges reported by Holmes *et al.* [38] included lack of physical exercise; financial impact of quarantine; and feeling pressure to continue working. In addition to these emotional and psychological impacts, participants noted experiencing sleep disturbances, including nightmares, which were linked to heightened anxiety [37]. Some individuals highlighted the challenges of boredom and loneliness, which were pervasive during self-isolation and compounded feelings of isolation and disconnection [32,34,37,38]. Furthermore, a sense of uncertainty about the future and their circumstances added to the psychological burden [32].

One quantitative study found that an independent measure of resilience mediated a decrease in anxiety [35], whereas another study found no association between resilience and wellbeing [38]. Additionally, positive opinions on pandemic prevention were found to be associated with resilience in one study [38]. Qualitative participants described coping strategies which were perceived to make self-isolation easier, including religious practices [34]; support from others [32,33,35,37]; exercise [35]; keeping a positive mindset [35]; doing activities relating to work while in self-isolation, such as reading relevant literature [33]; and self-care activities [35,37].

## DISCUSSION

The present review summarized and appraised the available literature assessing the impact of self-isolation on HCWs psychological wellbeing during the COVID-19 pandemic. Overall, the included studies represent a range of isolation contexts, healthcare disciplines and countries. Quantitative outcomes and evidence were heterogenous, and of ‘high’ or ‘very high risk of bias’, with an inconsistent trend towards an increase in anxiety, depressive and stress symptoms. The qualitative studies were of satisfactory methodological quality and indicated that while multiple factors negatively affected HCWs’ wellbeing during isolation, there were also factors that improved and protected wellbeing. Finally, no studies assessed the efficacy of interventions to increase HCWs’ wellbeing during isolation, highlighting an important area of research during future infectious disease outbreaks.

Across methodologies, our results cumulatively suggest that self-isolation had a negative impact on HCWs’ wellbeing during the COVID-19 pandemic. Quantitative studies primarily focused on psychopathology using self-report measures, with most studies assessing anxiety, depressive and stress symptoms. However, these scales often fail to capture broader aspects of wellbeing, such as frustration and agitation, which are critical for understanding factors such as government mistrust and reduced adherence to mitigation strategies [41,42]. Furthermore, although it was rarely reported and not formally extracted, HCWs’ isolation may have been employer-mandated, introducing unique workplace-related dynamics that could influence satisfaction and fulfilment. Indeed, we anticipated that occupational exposures and isolation would likely result in adverse outcomes such as moral injury in HCWs, but none of the studies included in our review assessed this. Qualitative evidence showed that many participants reported negative psychological effects, most prominently fear (e.g. of the disease or potentially spreading it) and loneliness, several studies also reported evidence of potential post-traumatic growth (e.g. renewed sense of gratitude and new perspective on patient care). As such, despite reports of several factors which were perceived to make the situation more challenging (e.g. stigma, financial stress, etc.) there were also factors perceived to make self-isolation easier (e.g. support from others).

These findings align with those reported in a previous review of the general population [2]. In the present review and that of the general population, qualitative studies provided a more nuanced understanding of self-isolation’s impact on wellbeing, capturing broad domains such as fear (present review) and worry (general population review) [2]. Protective factors were identified in both reviews, with social support and coping strategies serving as key buffers for both HCWs and the general population. However, a notable discrepancy emerged between the current review and that of the general population: while PTSD symptoms were a consistent concern in the general population, our review could not synthesize PTSD symptoms as a psychological outcome due to the limited number of available studies (*n* = 1). Additionally, protective factors were more comprehensively explored in our review compared to that of the general population. This discrepancy is plausibly due to the availability of primary research within each review and highlights the need for further research to better characterize PTSD-specific risk to HCWs, ensuring a more comprehensive understanding of the psychological impact of self-isolation.

The results of this review should be interpreted in light of the strengths and limitations of both the included articles and the review process itself.

All the quantitative studies were rated as ‘high’ or ‘very high risk of bias’. Most studies (*N* = 12) showed at least ‘some concerns’ regarding the selection of sample participants, and all studies were rated as having at least ‘some concerns’ related to bias in their selected results. This was typically due to a lack of pre-registration. During future infectious disease outbreaks, researchers should prioritize pre-registering their study protocols and planned analyses to enhance transparency and reliability. Similarly, three of the quantitative findings relied on study-specific measures (e.g. four-item Likert sleep scale, five-item Likert assessing needs, personal resources and workplace satisfaction, and unvalidated PTSD measure) which limited the reliability and validity of their results. Future studies should prioritize the use of established, validated measures. Finally, the quantitative results were mixed, with no outcome domain reaching consensus. This variability may be attributable to study-specific contexts. For instance, one study assessing sleep quality found that isolation improved sleep [28], likely reflecting the sample’s specific characteristics, where pre-isolation working conditions had adversely affected sleep duration and quality. Future studies should consider and clearly report contextual factors, such as pre-isolation conditions, to improve comparability and generalizability of findings across diverse populations.

Qualitative studies were of satisfactory methodological quality overall, but there were several items on the quality appraisal checklist where studies most often lost points. First, none of the studies discussed reflexivity, or considered how the researchers’ own expectations, perceptions or biases may have influenced data collection or analysis. Second, in at least four studies it was unclear whether data analysis was sufficiently rigorous, and several studies did not describe their analytic procedure in enough detail for the study to be replicable. Finally, it was unclear for most studies whether recruitment strategies were appropriate. Future research should aim to address these limitations and ensure that they are clearly discussed within the text.

No studies in this review assessed the efficacy of interventions, psychological or practical, aimed at improving HCWs’ wellbeing during isolation. This represents a significant gap in the literature, as targeted interventions will play a crucial role in mitigating the psychological and emotional challenges faced by HCWs during future infectious disease outbreaks. The absence of intervention studies may reflect structural barriers such as stigma, confidentiality concerns, limited resources and the need for leadership support [43]. In the context of a pandemic, this gap may reflect institutions’ lack of preparedness, as they were responding to a rapidly evolving crisis without the pre-planning or resources needed to support protective measures [44]. Addressing this gap through rigorous research is essential to inform evidence-based strategies that enhance HCWs’ resilience and wellbeing during future infectious disease outbreaks.

Our review process had many strengths, such as a pre-registered protocol, no geographic or language limitations, comprehensive risk of bias assessment and adherence to PRISMA, SWiM and eMERGe guidelines. However, we included the CASP checklist for completeness, though its generality limits its usefulness as a quality appraisal tool in qualitative synthesis, reflecting wider debates about quality assessment in qualitative research [45]. Human error may have resulted in missed studies during the selection process. Similarly, while data extraction was discussed with the review team, the absence of a dual-coder process may have increased the risk of errors.

This review provides a summary of the current evidence on the impact of self-isolation on HCWs’ psychological wellbeing during the COVID-19 pandemic. While qualitative findings highlighted both challenges and growth opportunities during self-isolation, quantitative studies presented inconsistent evidence, trending towards negative psychological effects such as increased anxiety, depressive and stress symptoms. The absence of research on interventions to improve HCWs’ wellbeing during self-isolation represents a critical gap that should be addressed before future infectious disease outbreaks. Public health strategies should prioritize timely, clear communication, accessible psychological and practical support, and resources to mitigate psychological harm. Future research should address methodological limitations in the current evidence base by incorporating pre-registration, validated measures, and workplace-specific stressors and outcomes to enhance reliability and relevance. By closing these gaps, research can provide actionable insights to better support HCWs and enhance their psychological wellbeing during public health crises.

## Data Availability

The data are freely available in all included articles. Extracted data can be freely accessed: https://osf.io/jngme/.
